# Feasibility of a Shape-Memory-Alloy-Actuator System for Modular Acetabular Cups

**DOI:** 10.3390/bioengineering11010075

**Published:** 2024-01-12

**Authors:** Christian Rotsch, Karoline Kemter-Esser, Johanna Dohndorf, Marcel Knothe, Welf-Guntram Drossel, Christoph-Eckhard Heyde

**Affiliations:** 1 Fraunhofer Institute for Machine Tools and Forming Technology IWU, 01187 Dresden, Germany; karo.ke@outlook.com (K.K.-E.); johanna.dohndorf@tu-dresden.de (J.D.); welf-guntram.drossel@iwu.fraunhofer.de (W.-G.D.); 2 University of Leipzig Medical Center, Orthopaedic, Trauma and Plastic Surgery Clinic, University of Leipzig, 04103 Leipzig, Germany; christoph-eckhard.heyde@medizin.uni-leipzig.de; 3 Institute of Biomedical Engineering, TUD Dresden University of Technology, 01307 Dresden, Germany; 4 IMA Materialforschung und Anwendungstechnik GmbH, 01099 Dresden, Germany; marcel.knothe@ima-dresden.de; 5 Professorship Adaptronics and Lightweight Design in Production, Chemnitz University of Technology, 09107 Chemnitz, Germany

**Keywords:** shape memory alloy, Nitinol, NiTi, SMA, implant, hip implant, acetabular cup, extraction, implant revision

## Abstract

Hip implants have a modular structure which enables patient-specific adaptation but also revision of worn or damaged friction partners without compromising the implant-bone connection. To reduce complications during the extraction of ceramic inlays, this work presents a new approach of a shape-memory-alloy-actuator which enables the loosening of ceramic inlays from acetabular hip cups without ceramic chipping or damaging the metal cup. This technical in vitro study exam-ines two principles of heating currents and hot water for thermal activation of the shape-memory-alloy-actuator to generate a force between the metal cup and the ceramic inlay. Mechanical tests concerning push-in and push-out forces, deformation of the acetabular cup according to international test standards, and force generated by the actuator were generated to prove the feasibility of this new approach to ceramic inlay revision. The required disassembly force for a modular acetabular device achieved an average value of 602 N after static and 713 N after cyclic loading. The actuator can provide a push-out force up to 1951 N. In addition, it is shown that the necessary modifications to the implant modules for the implementation of the shape-memory-actuator-system do not result in any change in the mechanical properties compared to conventional systems.

## 1. Introduction

Modular systems are frequently used for hip implants. These components are combined based on the patient’s condition, including age, height, weight, bone quality, primary implantation, number of revisions, leg length, femoral offset, or femoral anteversion [1,2]. In addition to anchoring the implant in the bone, tribological parameters are a central aspect of functionality. Currently, there are two main concepts for hip implants. On the one hand, hard–soft pairing exists, usually consisting of a polyethylene inlay for the acetabular cup and a ceramic (CoP = ceramic on polyethylene) or metal ball (MoP = metal on polyethylene) for the hip stem. These systems are widely recognized, and manufacturers of implants offer various configurations.

On the other hand, hard–hard pairing involves a ceramic inlay or ceramic cup and ball (CoC = ceramic on ceramic). These systems come in various sizes and configurations and are considered standard for clinical treatment. They have shown promising results in laboratory tests and long-term studies. However, there are differences in wear parameters, particle size, implantation process requirements, and revision properties [1,2,3,4].

If a worn component (inlay and/or ball) that has sufficient osseointegration with the metal parts (hip stem and/or acetabular cup) is damaged, the worn parts can be replaced while considering the clinical and technical boundary conditions [5,6].

The extraction of the ceramic inlay in ceramic–ceramic pairings presents a challenge. Unlike the polyethylene inlay, the ceramic inlay cannot be deformed, and drilling or perforating it during extraction is very difficult. The current state-of-the-art method involves applying impact to the edge of the metal cup to loosen the inlay, typically using a hammer and chisel [7], a high-frequency drill at the outer diameter of the acetabular cup [8], a hammer and metal impactor after drilling a wedge into the cup [9], or pneumatic systems [10]. The resulting impact can lead to mechanical stress in the ceramic component, damaging the inlay and metal cups, resulting in ceramic splinters (“chipping”). Mechanical impact can also influence the implant–bone interface [11,12,13].

This technical in vitro study aimed to develop a proof-of-concept for an actuator system that can produce a push-out force between the ceramic inlay and metal cup during the revision process without damaging these components. The actuator should not influence the implant or its behavior during normal use in implanted situations. Therefore, it should be small, and additional components such as batteries, sensors, and cables must be avoided in its vicinity. To demonstrate mechanical compatibility with other systems, as described above, it is necessary to consider the applicable test standards for hip implants. In our study, we focused on ASTM F1820 [14] and ISO 7206-12:2016 [15] to analyze the influence of modified implant geometries on push-in and push-out forces and deformation behavior compared to state-of-the-art implants. External stimuli may only activate the actuator system during the surgical intervention. Previous studies conducted by our team [16,17,18] and results from other research groups [19,20,21,22,23,24,25] have shown the potential of shape memory alloys (SMAs), especially nickel–titanium (NiTi), as components for implant applications. NiTi shows sufficient biocompatibility compared to other implant materials, such as Titanium and Titanium alloys, nickel-free stainless steel, or magnesium alloys [26,27,28,29,30,31,32]. Depending on the requirements of the implants or components, NiTi can be coated, for example with hydroxyapatite [33] or diamond-like coating (DLC) [26], to improve corrosion resistance or biocompatibility. The SMA has the highest volume-specific workload compared to other actuator concepts such as electrical or pneumatic drives [34]. Therefore, SMA actuators are extremely small, and they can be activated by thermal stimulus applied by the surroundings (friction or waste heat) or resistance heating. The activation temperature (phase transformation temperature) depends on the alloy type. In our study, we used an alloy with an austenitic finish temperature over a body temperature of 37 °C to avoid accidental activation. SMAs allow a programming of their geometry and mechanical behavior by applying specific thermal mechanical treatments [35,36].

The actuator system presented in this study consists of an SMA actuator placed between the ceramic inlay and metal cup. An additional layer between the actuator and metal cup isolates and positions the SMA actuator. The cup surface contains connection points for external SMA activation. The thermal stimulus leads to the contraction of the actuator, causing a push-out force that loosens the press-fit connection, allowing for the inlay removal.

This system provides a new approach to ceramic inlay revision with less mechanical impact on the acetabular cup and the inlay than the described conventional methods. This reduces the risk of damaging implant components, influencing the implant–bone interface and entry of ceramic splinters into the wound. In addition to this safety improvement, it is easier to handle for the surgeon.

In our study, we present the basic system concept and demonstrate its feasibility in pre-clinical mechanical tests according to the standards ASTM F1820 and ISO 7206-12:2016, focusing on push-in and push-out forces, deformation of the acetabular cup, and force produced by the actuator.

## 2. Materials and Methods

### 2.1. Overview Implant Components

**Figure 1:**
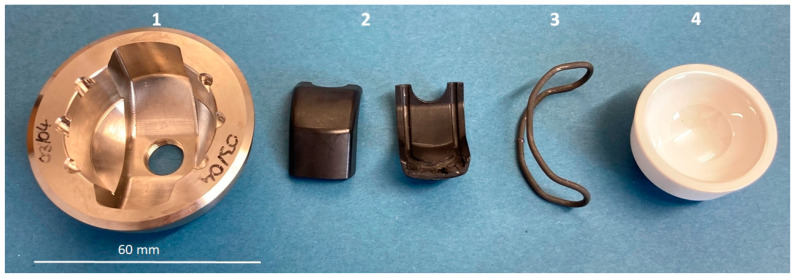
Implant components, overview. 1—acetabular cup, 2—PEEK device, 3—SMA-actuator, 4—ceramic inlay.

The final functional sample of the acetabular cup implant (Figure 1) consisted of a modified “Multicup II” metal cup made of a titanium–alumina–vanadium alloy (TiAl6V4 Grade 5) with an outer diameter of 58 mm and a modified inner geometry. Coating was not applied outside the acetabular cup because biomechanical experiments with biological or artificial bone materials were not planned for the prototype. The inner geometry of the acetabular cup was modified to integrate an additional polyether ether ketone (PEEK, TECAPEEK MT black, Ensinger GmbH, Nufringen, Germany) positioning device (acetabular cup and PEEK device; Aristotech Industries GmbH, Luckenwalde, Germany). PEEK is a biocompatible polymer used in a variety of implant applications [37,38].

A ceramic inlay (Mathys Orthopädie GmbH, Mörsdorf, Germany) made of Alumina Toughened Zirconia Ceramic (ATZ) “ceramys” was used. This material is commercially available and consists of 80% ZrO_2_ and 20% Al_2_O_3_ [39]. The inlay has an inner diameter of 32 mm and a modified shape adapted to the acetabular cup.

The PEEK device consisted of two parts that integrated and fixed the SMA actuator. In addition to the positioning of the actuator, the PEEK device was an insulation layer between the metal cup and the SMA actuator during the thermal activation process.

**Figure 2:**
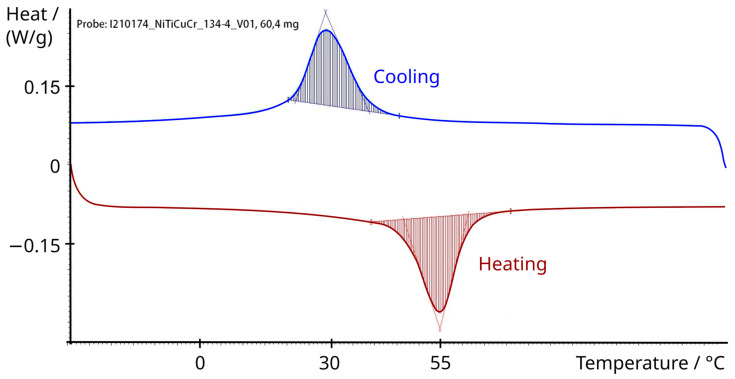
Differential scanning calorimetry (DSC) of NiTiCuCr alloy for the determination of the phase transformation temperatures.

The SMA actuator consisted of a Nickel–Titanium–Copper–Chromium (NiTiCuCr) alloy wire with a length between 130–136 mm and a diameter of 1.95 ± 0.015 mm (Ni42.5Ti49.9Cu7.5Cr0.1 alloy, Ingpuls GmbH, Bochum, Germany). The elements Copper and Chromium influence the phase transformation behavior compared to the binary alloy NiTi. The NiTiCuCr alloy exhibited an austenitic finish temperature of 54 °C (manufacturer specification). Therefore, the actuator had to be heated to at least 54 °C to complete the phase transformation. According to [40], a short-term heating to 54 °C should not lead to injuries of the surrounding tissue, especially considering that the SMA actuator is not in direct contact with the tissue. The SMA wire was laser-welded and formed into a loop, followed by a thermal mechanical process. To determine the transformation temperatures of the SMA wire after the thermal mechanical processing, a differential scanning calorimetry (DSC) analysis was performed (STARe System DSC, Mettler-Toledo GmbH, Gießen, Germany). The austenitic finish temperature of 54.35 °C and the martensitic finish temperature of 29.39 °C (Figure 2) confirm the manufacturer material specification.

**Figure 3:**
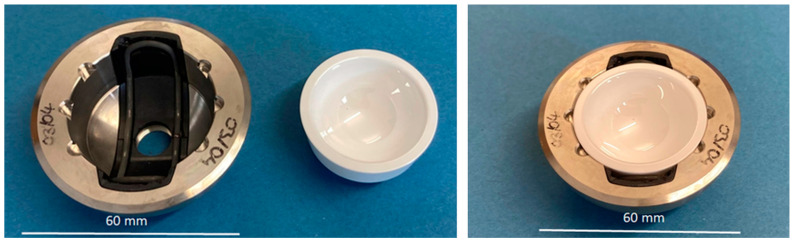
Implant components, assembly steps.

The assembly of the implant components was performed as follows. The PEEK device was positioned in the metal acetabular cup (Figure 3, left). Then, the SMA actuator was placed in the PEEK device, and the ceramic inlay was pushed in (Figure 3, right).

### 2.2. Shape Memory Alloy Actuator

We used a thermal one-way effect instead of the antagonistic principle for the functionality of the SMA actuator. The actuator changed its crystal structure by heating to the austenitic finish temperature and fulfilled a phase transformation. This led to a change in the outer geometry and the alloy was in an austenitic state [41,42]. The concept provides that the activation is only performed once to extract the inlay. After extraction, a new actuator and inlay should be used for further surgical intervention. For technical evaluation in this study, the actuator could be used several times.

**Figure 4:**
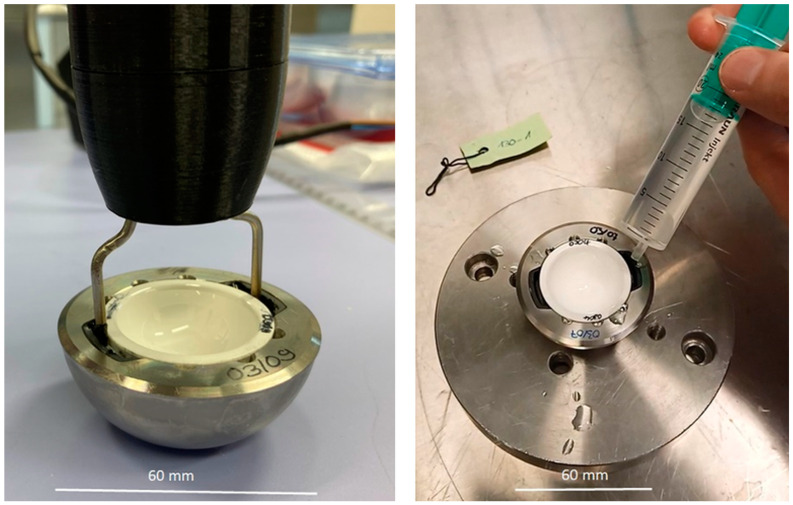
Activation of the SMA actuator with activation instrument (**left**) and with hot water (**right**).

Hot water applied with a water-filled syringe of approximately 70 °C (Figure 4, right) or resistance heating by an electrical current instrument (endocon GmbH, Wiesenbach, Germany) achieved thermal activation of the SMA actuator (Figure 4, left). The instrument consisted of a handle with a switch and a power supply with a controller based on an Arduino miniboard. The functional end of the instrument consisted of two electrical contacts connected to the actuator. A current up to 10 A can be applied with the present resistance of the actuator and a power supply of 7.4 V (TTi EX2020 R Power Supply, Turbly Thandar Instruments Ltd. Cambridgeshire, UK). The power supply time should not exceed 60 s for one activation.

With both thermal activation options, the pre-stretched actuator can be heated to phase transformation. Therefore, the actuator can push out the ceramic inlay. The actuator was used again after the push-out procedure. For this purpose, the actuator was cooled and pre-stretched again.

**Figure 5:**
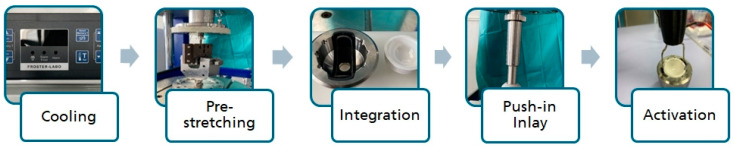
Process chain of the SMA actuator.

The process chain of the SMA actuator is shown in Figure 5.

### 2.3. Experimental Setup

**Figure 6:**
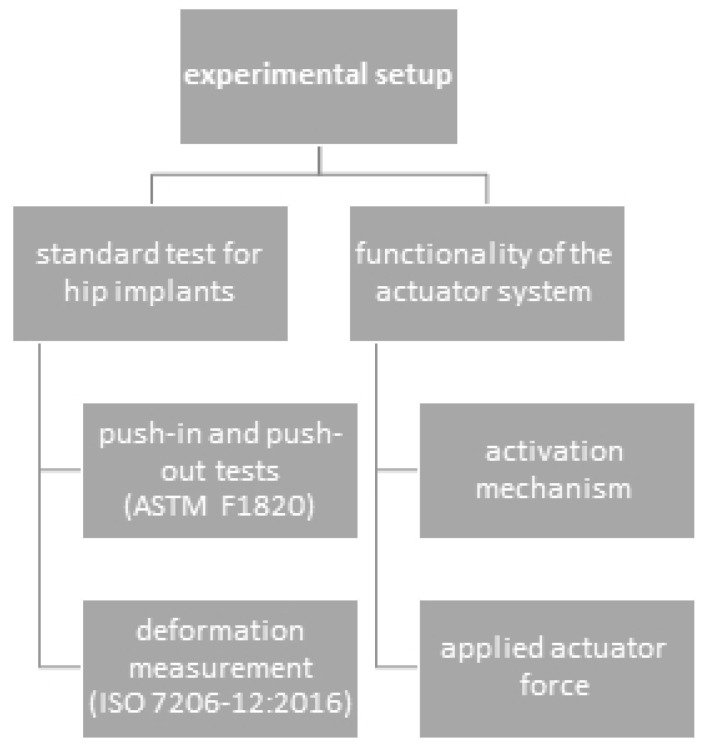
Experimental setup, scheme.

We pursued two strategies to demonstrate the feasibility and performed a preclinical evaluation of the SMA actuator system (Figure 6). First, we evaluated the influence of the modifications of the acetabular cup components on the mechanical behavior according to the selected test standards. These tests were conducted according to the standards ASTM F1820 “Push-out and torsion tests for acetabular cups” and ISO 7206-12:2016 “Deformation measurement of acetabular cups with and without ceramic inlay”. However, the aim was to evaluate the behavior of the actuator system with respect to the applied push-out force and actuator behavior. These tests were conducted using self-developed test rigs (see Figure 6).

The recorded push-in and push-out forces as well as the resulting actuator forces are defined as positive in the following chapters.

#### 2.3.1. Push-In and Push-Out Tests for Acetabular Cups

**Figure 7:**
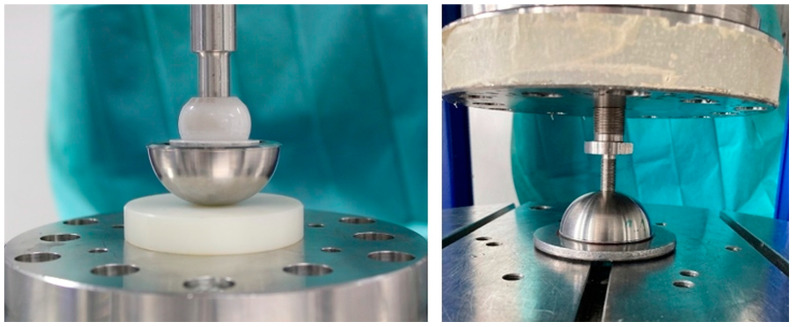
Push-in (**left**), push-out (**right**) of ceramic inlay.

Push-in and push-out tests of the ceramic inlay following ASTM F1820 were conducted with modified implant components consisting of a metal hip cup (original and modified shapes), ceramic and PEEK inlays, and SMA actuators with lengths between 130–134 mm. For this purpose, tests were performed using a tension-compression-torsion testing machine (see Section 2.2). The PEEK inlay and SMA actuator were placed in a titanium cup. A test stamp consisting of a ceramic ball and test pin was used to press the inlay into the hip cup, which was positioned on a polytetrafluoroethylene (PTFE) plate (Figure 7, left). The line of force applied was congruent with the pole axis of the inlay. Then the ceramic inlay was joined with a speed of 0.04 mm/s and a peak force of 2000 N. The joining process was possible without any limitations on the force or tilting of the inlay.

The push-out test was used to examine the possible influence of the modified implant geometry on the necessary push-out force. The axial force was applied to the inlay through the center hole (pole axis of the cup) using a rod with a diameter of 6 mm at a test speed of 0.04 mm/s until the inlay detached from the metal hip cup (Figure 7, right). Subsequently, the required force was measured.

**Figure 8:**
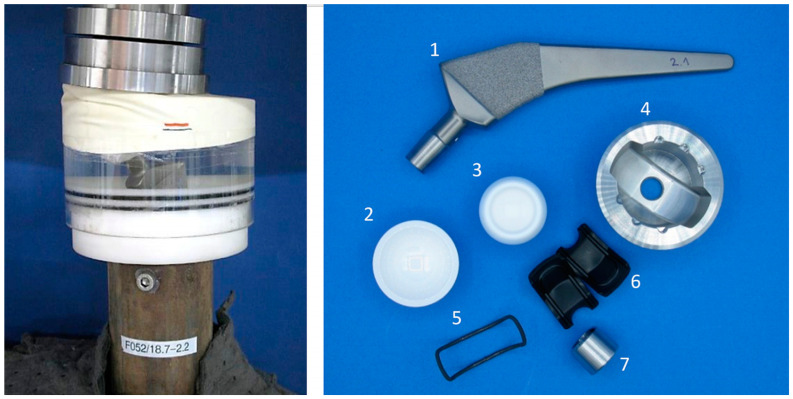
Test setup (**left**), implant components (**right**) for tests according to ASTM F 1875 [43] and ISO 7206-4:2010 [44]; 1—hip stem, 2—ceramic inlay, 3—ceramic ball, 4—acetabular cup, 5—SMA actuator, 6—PEEK device, 7—head adapter system.

To exclude the influence of multiple uses, the push-in and push-out tests were repeated with the new implant components. In further experiments, the influence of a dynamic load on the push-out forces was investigated and compared to the original hip cup shape. Therefore, we used modified acetabular cups for corrosion tests according to ASTM F1875 using a cyclic testing machine (self-made 16 kN fatigue machine, IMA Materialforschung und Anwendungstechnik GmbH, Dresden, Germany). During the tests, a force of 230 N to 2300 N was applied sinusoidally at 5 Hz over ten million cycles. The test setup and the implant components are illustrated in Figure 8.

#### 2.3.2. Deformation Measurement of Acetabular Cups with and without the Ceramic Inlay

The ARAMIS SRX 12M system (Carl Zeiss GOM Metrology GmbH, Braunschweig, Germany) was used for the determination of the deformation. ARAMIS is a non-contact 3D optical deformation measuring system providing analysis, calculation, and documentation. The measurement results were graphically displayed. In digital camera images, ARAMIS recognizes the surface structure of the measurement sample and assigns coordinates to image pixels. One image represented the initial state of the specimen. After loading, another image was taken. The result of the comparison of these digital images was the deformation of the specimen.

**Figure 9:**
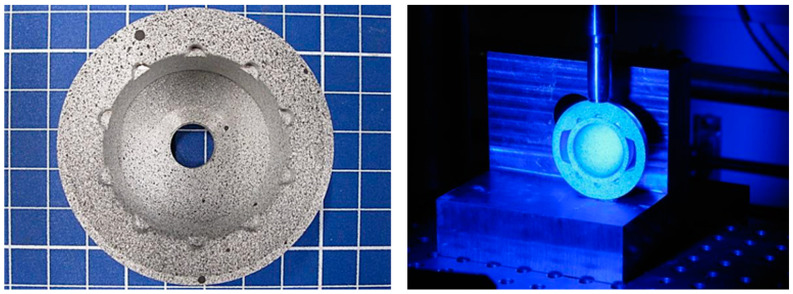
Hip cup with spray patter (**left**), test setup hip cup deformation (**right**).

In the test setup, an L-profile supported and positioned the acetabular (Figure 9, right). The specimens were subjected to a diametrically opposed two-point loading. Measurements of the diameter in the direction of loading were performed in a defined measuring plane before, under loading, and after unloading to determine the short-term deformation. This measurement was repeated twice after the specimen had rotated to consider the influence of asymmetrical design features such as ribs or holes. The angular positions of the three measurements in the original cups were 0°, 120°, and 240°. Angular positions of 0°, 90°, and 135° were selected to account for the influence of the recess in the modified cups. The load was applied collinearly and orthogonally to the recess in the 0-degree and 90-degree positions, respectively.

Because the edges of the pans were quite homogeneous, that is, they had only a few object features, the surfaces were pre-treated with a stochastic color spray pattern (Figure 9, left).

The ceramic inlay significantly influenced the deformation properties; hence, cups without inlays and those with properly inserted and joined inlays were both tested. For this purpose, the inlays were mounted in a cup with a peak force of 2000 N using a compression-torsion testing machine (MTS 858 Mini Bionix II, Eden Prairie, MN, USA). The tests were evaluated by comparing the graphical representations of the deformation and maximum changes in the diameters.

The permanent deformation is defined according to ISO 7206-12:2016 as:

(1) $$\left|\frac{D_{0^{\prime }}-D_{0}}{D_{0}-D_{1}}\right|>0.02$$

D_0_—Diameter before loading;

D_1_—Diameter under loading;

D_0^′^_—Diameter after loading/unloading.

#### 2.3.3. Functionality of the Actuator System

**Figure 10:**
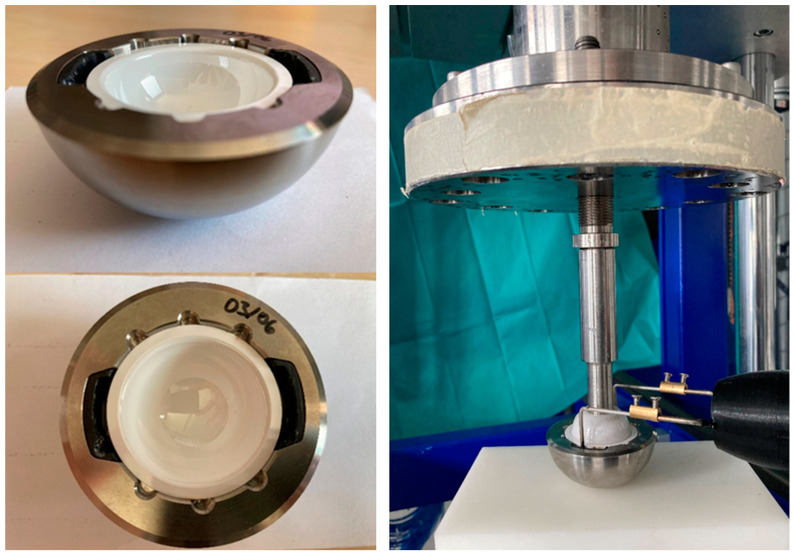
Ceramic inlay with undersize (**left**), instrument and implant components in the experimental setup (**right**).

Static tests were performed using a modified ceramic inlay with a diameter reduced by 0.6 mm (Mathys Orthopädie GmbH, Mörsdorf, Germany) to determine the resulting actuator forces. The modification prevented conical clamping between the inlay and cup Figure 10, left), which enabled the recording of the actuator forces on the ceramic inlay with a compression-torsion testing machine (see Section 2.2). The test stamp, consisting of a ceramic ball and test pin, moved to contact with a pre-force of approximately 10 N and was held in position. The SMA actuator was then activated by an electrical current using an instrument (Figure 10, right) (endocon GmbH, Wiesenbach, Germany). The resulting compression forces were recorded as a measure of actuator-generated forces.

**Figure 11:**
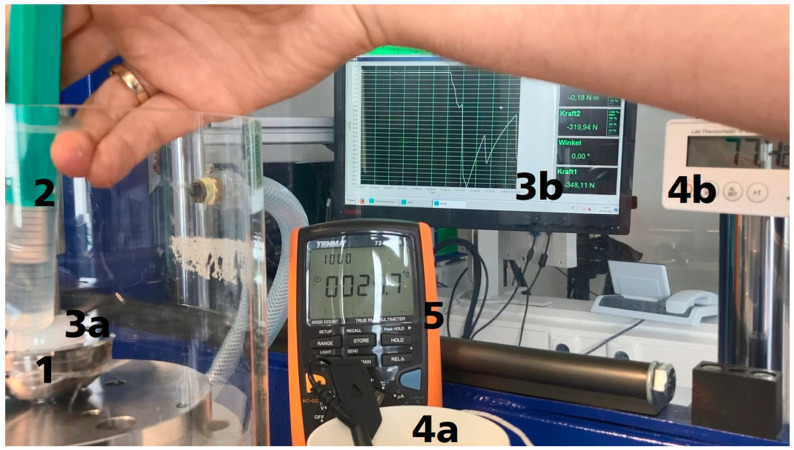
Test setup for evaluating the actuator force with heated water: 1—hip cup with ceramic inlay, PEEK inlay with actuator, 2—syringe with hot/cold water, 3a—test setup for determining the resulting actuator force, 3b—display of the test machine, 4a—cup with water, 4b—temperature display for water, 5—display temperature sensor attached to the outside of the metal cup.

Activation using hot water was also examined as an alternative to electrical activation. This allowed the exclusion of insufficient electrical contact and increased contact resistance. Insufficient electrical contact can lead to an inhomogeneous warming of the actuator. This results in inadequate activation and actuator force. Activation with hot water could enable complete heating of the actuator. For this purpose, supplementary to the test setup shown in Figure 10, right, a waterproof poly methyl methacrylate (PMMA) cylinder was mounted on the testing machine. The inlay was joined again with −2000 N, as illustrated in Figure 7, left, and then loaded with a pre-load between 20 and 50 N. The inlay was heated and cooled cyclically using tempered water and a syringe. The temperature for cooling was between 21 °C and 24 °C and for heating it was between 70 °C and 80 °C. This is higher than the sufficient temperature of 54 °C for phase transformation according to alloy composition. Thus, influences due to non-optimal electrical contact and an incomplete phase transformation can be excluded. A probe thermometer (TFA, Dostmann GmbH & Co. KG, Wertheim-Reicholzheim, Germany) was used to measure the temperature of the water reservoir. A temperature sensor was placed on the outside of the metal cup and coupled with a multimeter (Tenma Test Equipment, Centerville, OH, USA) to measure the change in implant temperature due to the applied water. The resulting forces, water temperatures, and heating outside the cup were recorded. The test setup is shown in Figure 11.

## 3. Results

### 3.1. Push-In and Push-Out Tests for Acetabular Cups

#### 3.1.1. Push-In Tests

The push-in tests results are listed in Table 1. A peak force of 2000 N, according to ASTM F1820, was reached in all experiments, with slight deviations owing to the control parameters of the testing machine.

**Figure 12:**
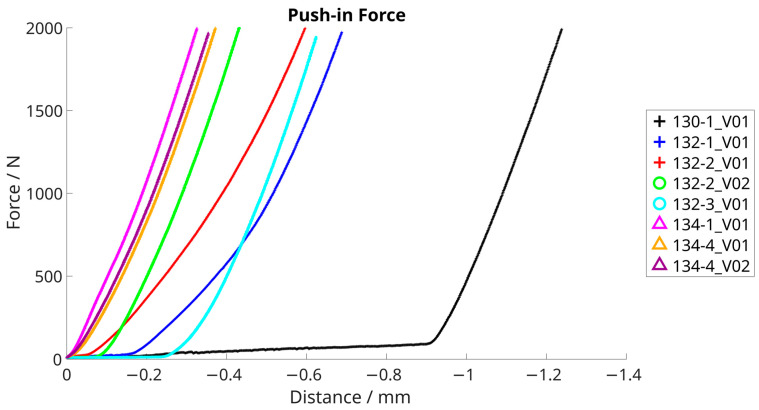
Push-in of ceramic inlays, force–distance diagram.

The force–distance diagram is shown in Figure 12. It is used to visually analyze the force curve during joining. There are no force peaks or irregularities in the curve that would indicate jamming or a faulty joining. The different distances are a result of the different initial positions of the testing machine. The decisive factors are the comparable curves and the forces achieved from the contact between the machine and the specimen.

#### 3.1.2. Push-Out Tests

In a first series (A), the actuator device was varied to investigate the influence of different actuator lengths. The necessary forces to push the inlay out of the acetabular cup are listed in Table 2. Their mean value is 602 N with a standard deviation of 304 N, and in all tests except one (134-1_V01 with 306 N), the forces are greater than 498 N.

It should be noted that all the components were used several times in the push-in and push-out tests.

**Figure 13:**
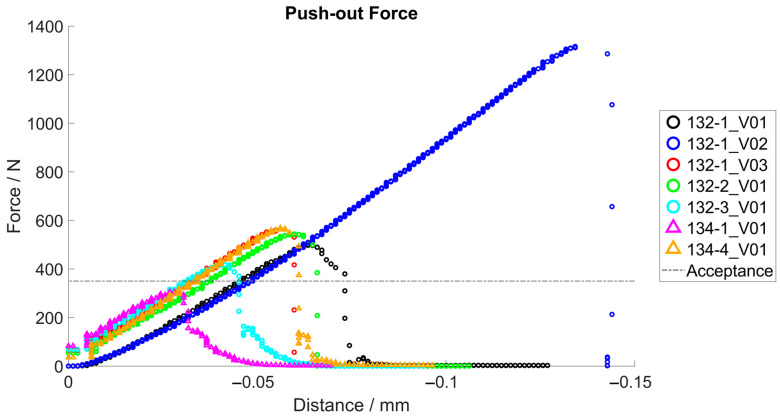
Push-out force, force–distance diagram.

The relative position of the testing machine and the resulting force are shown in Figure 13. It is used to visually analyze the force curve during push-out tests. There are no force peaks or irregularities in the curve that would indicate jamming or problems during the removal process. The increases in the curves are comparable.

In a second test series, new implant components (inlay and acetabular cup) were used to analyze the influence of the number of test cycles. The modified and original cup geometries were used for comparison. The same actuator was used for each repetition of the modified shape. These tests were performed three times. In Table 2, the results of the test repetitions with new components are shown. After static loading, the modified cups (B) reached a push-out force of 630 N on average, slightly lower than the original shape cups (C) with 670 N. After the cyclic loading, the modified cups (D) reached an average push-out force of 683 N. This value was slightly lower than that of the original cup, which reached an average of 713 N after cyclic loading (E). These tests were performed only two times. The calculation of the standard deviation was not applicable.

The modified and original shape components reached a minimum required push-out force of 350 N.

**Figure 14:**
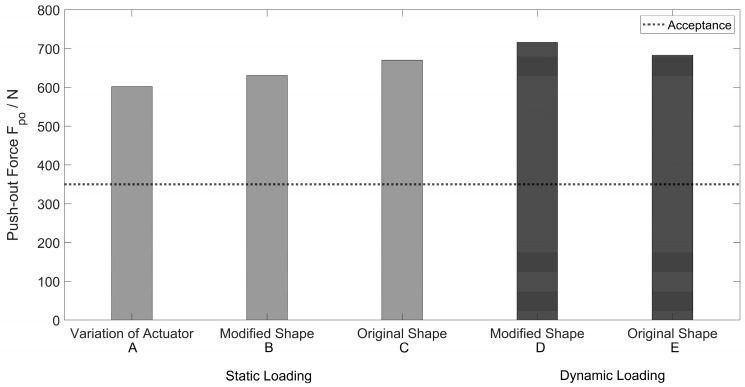
Results push-out tests.

The results (mean values) of the push-out tests are shown in Figure 14. All push-out forces are higher than 600 N. The acceptance criterion of at least 350 N is fulfilled with the modified shape and the original shape components. A minimal push-out force of 350 N is based on experimental data from Steinhauser et al. [45] multiplied by a safety factor of 1.5.

### 3.2. Deformation Measurement of Acetabular Cups with and without the Ceramic Inlay

**Figure 15:**
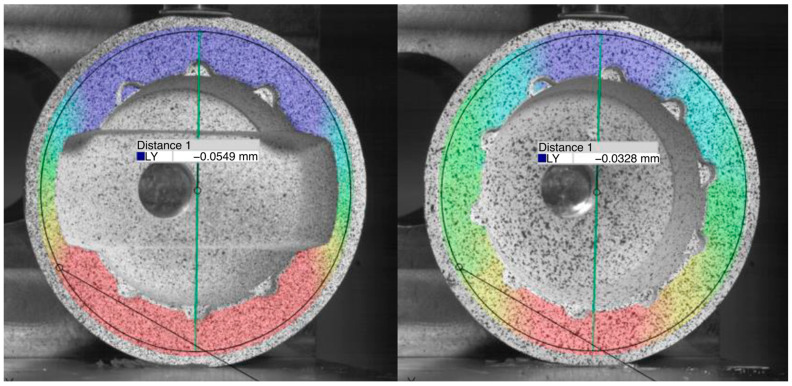
Deformation of metal cup; (**left**) modified geometry, (**right**) original geometry.

The deformations could have been measured with the ARAMIS system, but they were too minimal (Figure 15 and Table 3) to form a useful ratio according to Equation (1).

### 3.3. Functionality of the ACTUATOR system

#### 3.3.1. Activation with Electric Current

**Figure 16:**
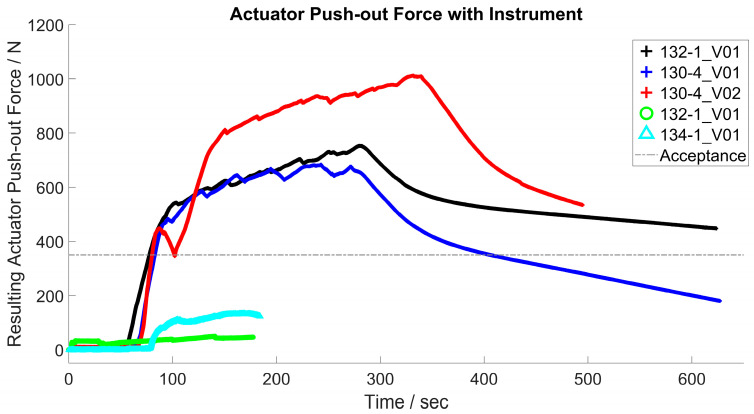
Forces generated by various actuators (130-1, 130-4; 132-1, 134-4) during push-out test, static state, heating by electrical current, acceptance criteria as reference.

Figure 16 shows exemplary force–time curves during activation with the instrument according to the principle in Figure 4, left. The different maximum forces during activation were striking. The power supply time was up to 300 s, including several short interruptions. During the tests, it was difficult to keep the electrical contact stable.

#### 3.3.2. Activation with Hot Water

**Figure 17:**
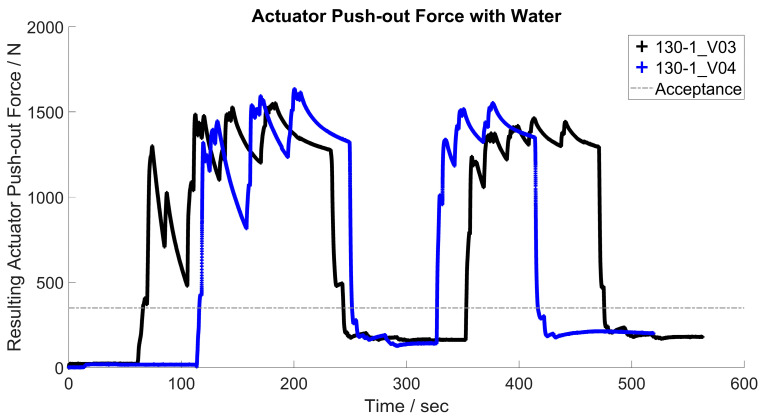
Test procedure for evaluation of the actuators 130-1 and 130-4, heating by water, acceptance criteria as reference (selection).

Figure 17 illustrates the temporal course of the activation experiment with hot water. An evident change in the resulting actuator force was observed during cooling and heating. The steeper increases in the curve in Figure 16 demonstrate faster heating (activation) and cooling (deactivation) of the actuators. The heating of the outside of the titanium hip cup was measured with approximately 10 Kelvin under lab conditions, starting with a room temperature of 22 °C and ending with a temperature of approx. 32 °C on the outside of the hip cup after one cycle of multiple warming.

Table 4 presents the maximum actuator forces determined during the cyclic tests. Actuators with a length of 130 mm exhibited considerably higher values than those with a length of 132 mm. Furthermore, higher forces were achieved compared to tests with electrical activation.

Table 5 shows the difference between activation by water and electrical current of the SMA actuator 130-1 and 130-4. Water activation led to an increase of approximately 1000 N resulting force.

## 4. Discussion

### 4.1. Push-In and Push-Out Tests

According to ASTM F1820, “Push-out and torsion tests for acetabular cups,” the push-out forces realized with the modified cups were 6% lower than standard geometries after static loading and 4.2% lower after dynamic loading. However, the internal acceptance criterion of the manufacturer (Mathys Orthopädie GmbH, Mörsdorf, Germany) with a push-out force of 350 N was achieved in all tests with new implant components.

For the experiments with implant components used several times in the push-in and push-out tests, only specimen 134-1_V01 did not reach the acceptance criteria. Sample 132-1 showed considerably different results in test V02 compared to tests V01 and V03. Incorrect positioning of the ceramic or peek inlay during joining could be a possible cause. The result of 132-1_V02 led to the large standard deviation of 304 N.

On average, push-out forces between 602 N and 713 N were measured under several test conditions with modified shape and original shape implant components. The results of our study are comparable to those of other studies in terms of push-out forces of ceramic inlays.

Lee et al. showed in a study push-out forces between 389.7 ± 108.3 N and 1148.8 ± 46.7 N depending on the insertion conditions and inner taper angle. The push-in forces were applied manually by an instrument [46].

In Hunger et al., push-out forces up to 932.27 N were measured, but the impaction force applied by a testing machine and an instrument was 4 kN [47]. McAuley et al. demonstrated that the higher joining force results in an increased push-out force [48].

The joining was performed at a force of 2000 N, and a maximum of 2200 N was achieved with a dynamic load. Higher joining forces resulting from manual joining, or loads on the implant during its service life, were not considered. The joining force of 2000 N was selected according to the test standards for acetabular implants to enable comparison and evaluation under standard conditions in an in vitro test environment. The additional components (PEEK inlay and SMA actuator) did not influence the joining of the titanium cup and the ceramic inlay. A considerable influence of the actuator system and geometric modifications on the mechanical behavior of the investigated implant components could not be determined from the tests performed.

### 4.2. Deformation Measurement of Acetabular Cups with and without the Ceramic Inlay

The tests conducted according to ISO 7206-12:2016 “Deformation measurement of acetabular cups with and without ceramic inlay” show that modifications of the titanium cup have a minor influence on the mechanical strength. The modified acetabular cup deformations were slightly greater than those of the standard cup. This applies to the tests with and without an inlay. Overall, the deformations were minimal. However, the determination of the permanent deformation according to ISO 7206-12:2016 cannot provide meaningful values because the deformations were too small to form a useful ratio. The increase in the modified cups’ deformation compared to the standard cups in the presented study had no influence on the mechanical stability of the metal hip cup under laboratory conditions.

The cup with the original shape and ceramic inlay showed the smallest deformation with 16.5 µm, while the highest deformation was shown by the modified cup without inlay with 55.2 µm. The ceramic inlay reduced the deformation nearly by half in both configurations. The deformations in our study are comparable to those found in previous research.

Vogel et al. investigated the deformation of the acetabular cup by varying the inlay material in a press fit scenario using an artificial bone model. This finite element analysis study calculated radial deformations of the titanium cup with a ceramic inlay up to 2 µm and with an ultra-high-molecular-weight polyethylene inlay (UHMWPE) up to 149.0 µm [49].

In their experimental study, Meding et al. analyzed the influence of acetabular cup design on deformation behavior. They measured initial cup deformations ranging from 8 µm to 267 µm [50].

### 4.3. Functionality of the Actuator System

The basic principle of thermal actuator activation worked as presumed. However, insufficient and unstable contact between the instrument and actuator led to fluctuations in the actuator forces achieved. The slow heating of the actuators was also noticeable. The force increased steadily and not abruptly. This also applied to the cooling of the actuators, which only took place through the ambient air. The required push-out forces regarding the acceptance criteria of 350 N were achieved in the successfully conducted tests, but the applied actuator forces seemed to be too low for a reliable extraction of the ceramic inlay compared to the push-out force measurements in Section 3.1.2.

The actuators 130-1_V01 and 130-4_V01 showed insufficient activation, resulting in the low increase in force. This could be a result of inadequate electrical connection or placement of the actuator. According to the actuator design and possible arithmetic stress, the measured actuator forces were too low. With a diameter of 2 mm and a possible actuator tension between 200 MPa and 800 MPa for non-cyclic applications, an actuator force between 630 N and 2500 N per wire section should be possible. Concerning the actuator design, a force between 2600 N and 10 kN should be achievable under uniaxial stress conditions. In practice, a load of approximately 6000 N is achievable.

In summary, the instrument used was not ideal for activation and electrical contact could not be stably established during practical tests. Minimal movement led to increased contact resistance and, presumably, inhomogeneous heating. Further investigations into electrical contact and energy input are necessary but could not be conclusively conducted during the study. The electrical and thermal conductivities of welded joints should be further investigated, too.

Alternative activation using warm water has proven to be practical and easy to apply under laboratory conditions. The increase of approximately 1000 N resulting force could be the result of the homogeneous and rapid heating by hot water. During this process, the outer side of the acetabular cup was heated to 10 Kelvin as the worst-case scenario. The heating at 10 Kelvin was comparable to bone cement (PMMA). The temperature reached in vivo was generally lower than the in vitro results owing to heat conduction in the implant and blood flow [48]. In combination with a small amount of water, 5–10 mL, this should not pose any additional problems in terms of protein denaturing and thermal tissue damage compared to bone cement, even for future in vivo applications.

However, tests on biomechanical models and, in the future, on animal models should be carried out to investigate the temperature distribution during activation. The SMA actuator was actuated and cooled several times during each experiment. The applied forces were reproducible. From the authors’ point of view, integrating a closed rinsing system in an instrument is a possibility for further optimization if syringe application proves inapplicable from a clinical perspective.

The design of the developed actuator appeared suitable for integration into acetabular implants. The developed PEEK inlay was functional and can support actuator forces. The actuator can generate the necessary force to release the conical clamping of the cup and inlay after the push-in at a maximum joint load of 2000 N. Due to the design and cross section of the actuator, forces of up to 6000 N should be achievable. However, this was not demonstrated in the tests conducted. Hence, the influence of pre-load and position variation of the actuators should be investigated in further studies.

The influence of dynamic and higher loadings during walking, running, or stumbling [51,52] were not focused on in this study and should be analyzed in further studies. It cannot be ruled out that higher dynamic loads under realistic conditions will also result in higher push-out forces. Consequently, it must be analyzed whether the actuator can reliably realize these push-out forces. From a theoretical point of view, the actuator concept should be capable of fulfilling these further requests.

In summary, the feasibility and pre-clinical technical evaluation of a shape-memory-alloy-actuator system for extraction of ceramic inlays from acetabular cups could be demonstrated in this technical study under in vitro test conditions. The developed SMA actuator system can generate the necessary push-out forces for the extraction of inlays after static loading up to 2000 N and cyclic loading up to 2200 N according to standard in vitro test conditions. Other studies showed push-out forces of up to 1148.8 ± 46.7 N after static loading with 2000 N and metal cups [46]. The highest actuator forces performed sample 130-1 and sample 130-4 with an average of 1630 N (peak force: 1805 N) and 1656 N (peak force: 1951 N) by heating up with hot water. The actuator forces would be sufficient for these push-out forces from the literature.

Although the materials used in the in vitro study are already established for implants (TiAl6V4 Grade 5, PEEK, SMA/NiTi, ATZ ceramic “ceramys”), the long-term behavior and the interaction of the materials (for example, corrosion, particle abrasion) should be analyzed, for example in cell biological studies or corrosion tests [53,54,55].

## 5. Conclusions

The actuator system based on SMAs can be integrated into a modified acetabular cup along with an additional PEEK device for isolation and fixation. The mechanical behavior of the system was not affected by the modified acetabular cup, the additional PEEK device, or the actuator, as confirmed by the applied tests.

The actuator system was able to generate the required push-out force to release ceramic inlays after static and dynamic loading.

Two methods were tested to induce a phase transformation of the SMA by heating, which resulted in the actuator push-out force. The electrical activation device showed less sufficient phase transformation due to unstable electrical contact. The second water-based method showed higher push-out forces and faster heating/cooling rates. Due to the unique conditions of the medical use case, the water-based solution appears to have better usability and higher intraoperative safety. However, to improve the instrument, it is necessary to integrate a closed rinsing system. Furthermore, it is important to study the biological interactions of the instrument.

Additionally, further investigations are required to analyze the mechanical long-term behavior, optimize the design of the SMA actuator, and improve the manufacturing process, including the welding and thermal mechanical shaping processes.

## Figures and Tables

**Table 1:** Push-in tests.

Push-in Force F_pi_ [N]		
	Sample *	Max
	132-1_V01	1998
	132-2_V01	1974
	134-4_V01	1998
	132-3_V01	1998
	134-1_V01	1944
	134-4_V02	1996
	132-2_V02	1968
	130-1_V01	1997
mean value	1984	
standard deviation	19	

* The nomenclature of the samples consists of the initial length of the actuator wire in mm (130 to 134), the consecutive number within an actuator length (1 to 4), and the number of trials (V01 to V04).

**Table 2:** Push-out test after static loading, influence of actuator length and implant component.

	Push-out Force F_po_ [N]					
	*static loading*					
	*modified Shape*				*original Shape*	
	sample * (A)	max	sample ** (B)	max	sample ** (C)	max
	132-1_V01	498	185-1_V01	578	185-2_V01	582
	132-1_V02	1316	185-1_V02	657	185-2_V02	696
	132-1_V03	560	185-1_V03	655	185-2_V03	733
	132-2_V01	546	-	-	-	-
	132-3_V01	417	-	-	-	-
	134-1_V01	306	-	-	-	-
	134-4_V01	569	-	-	-	-
mean value	602		630		670	
standard deviation	304		37		64	
	*cyclic loading*					
	*modified shape*		*original shape*			
	sample ** (D)	max	sample ** (E)	max		
	188-1_V01	651	188-2_V01	701		
	188-1_V02	774	188-2_V02	665		
mean value	713		683			
standard deviation	not applicable		not applicable			

* The nomenclature of the samples consists of the initial length of the actuator wire in mm (132 to 134), the consecutive number within an actuator length (1 to 4), and the number of trials (V01 to V04). ** The nomenclature of the samples consists of loading (185: static, 188: dynamic load), implant components (1: modified, 2: original shape), and number of trials (V01 to V03).

**Table 3:** Deformation measurements, hip cup.

	Deformation d [mm]		
sample	under loading	after loading	difference
original shape, without inlay	0.0301	0.0015	0.0286
original shape, with inlay	0.0165	0.0011	0.0154
modified shape, without inlay	0.0552	0.0008	0.0544
modified shape, with inlay	0.0280	0.0036	0.0245

**Table 4:** Actuator, max. push-out force, static (selection).

Push-Out Force Actuator F_po*_ [N]			
sample	max.	mean value	standard deviation
130-1	1805	1630	117
130-4	1951	1656	211
132-1	1073	987	89
132-3	1083	907	122

**Table 5:** Comparison between activation by water and electric current (selection).

Push-out Force Actuator F_po**_ [N]			
sample	water	electric current	difference
130-1	1805	753	1052
130-4	1951	1013	938

## Data Availability

The data presented in this study are available on request from the corresponding author.
